# Adherence and Associated Factors of Treatment Regimen in Drug-Susceptible Tuberculosis Patients

**DOI:** 10.3389/fphar.2021.625078

**Published:** 2021-03-15

**Authors:** Sungho Bea, Hyesung Lee, Ju Hwan Kim, Seung Hun Jang, Hyunjin Son, Jin-Won Kwon, Ju-Young Shin

**Affiliations:** ^1^ School of Pharmacy, Sungkyunkwan University, Suwon, South Korea; ^2^ Division of Pulmonary, Allergy, and Critical Care Medicine, Department of Medicine, Hallym University Sacred Heart Hospital, Hallym University, Anyang, South Korea; ^3^ Department of Preventive Medicine, College of Medicine, Dong-A University, Busan, South Korea; ^4^ BK21 FOUR Community-Based Intelligent Novel Drug Discovery Education Unit, College of Pharmacy and Research Institute of Pharmaceutical Sciences, Kyungpook National University, Daegu, South Korea; ^5^ Samsung Advanced Institute for Health Sciences and Technology (SAIHST), Sungkyunkwan University, Seoul, South Korea

**Keywords:** adherence, drug-susceptible tuberculosis, nationwide study, discontinuation, claims database

## Abstract

**Background:** Adherence to tuberculosis (TB) drugs is one of the key aspects of global TB control, yet there is a lack of epidemiological evidence on the factors influencing adherence to TB drugs. Thus, this study aimed to explore the adherence and factors associated with adherence among TB patients in South Korea.

**Methods:** We conducted a cohort study using a sampled national healthcare database from 2017 to 2018. Our study population included incident TB patients initiating quadruple or triple regimen who were available for follow-up for 180-days. Adherence was evaluated using the proportion of days covered (PDC): 1) adherent group: patients with PDC ≥80%; 2) non-adherent group: patients with PDC <80%. Kaplan-Meier analysis was conducted to calculate the median time-to-discontinuation in the study population. We calculated the adjusted odds ratios (aOR) with 95% confidence intervals (CI) to assess factors associated with adherence to TB drugs using logistic regression.

**Results:** Of 987 patients, 558 (56.5%) were adherent and 429 (43.5%) were non-adherent, with the overall mean PDC of 68.87% (standard deviation, 33.37%). The median time-to-discontinuation was 113 days (interquartile range 96–136) in the study population. Patients initiating quadruple regimen were more likely to adhere in comparison to the triple regimen (aOR 4.14; 95% CI 2.78–6.17), while those aged ≥65 years (aOR 0.53; 95% CI 0.35–0.81), with a history of dementia (aOR 0.53; 95% CI 0.34–0.85), and with history of diabetes mellitus (aOR 0.70; 95% CI 0.52–0.96) were less likely to adhere to the drug.

**Conclusion:** Approximately 45% of TB patients were non-adherent to the drug, which is a major concern for the treatment outcome. We call for intensified attention from the authorities and healthcare providers to reinforce patients’ adherence to the prescribed TB drugs.

## Introduction

Tuberculosis (TB) is an infectious disease caused by *Mycobacterium tuberculosis*, accounting for more than 10 million cases and 1.3 million deaths annually (WHO, 2018; Furin et al., 2019). Although less than 10 incident cases per 100,000 population were reported in most high-income countries, the disease burden from TB has been particularly high in South Korea, with average annual incidence rate of 70 cases per 100,000 population (KCDC, Division of TB & HIV/AIDS Control, 2018). To eradicate this prehistoric scourge by the year 2035, the World Health Organization established “The TB End Strategy” with the concept of high-level commitment and financing (WHO, 2014). Similarly, the Korean government launched “Public-Private Mix” in 2007 to provide supervision and patient support to improve adherence to the standardized TB treatment (Kim and Yim, 2015). However, despite these efforts, burden of TB in South Korea is the highest among the OECD member countries.

Adherence to TB drugs is critical in achieving successful treatment outcomes, controlling the spread, and preventing the development of drug resistance in TB. Completion of 2-months intensive followed by 4-to 7-months continuation phases of therapy has shown to cure most of the drug-susceptible TB cases with only less than 5–8% chances of relapse (Horsburgh et al., 2015), whereas non-adherence to TB drugs likely led to a multidrug-resistant TB (MDR-TB) or post-TB sequelae (Cox et al., 2006; Fløe et al., 2018). However, given the lengthy treatment duration, ensuring adherence to TB drugs poses a challenge (Karumbi and Garner, 2015). Thus, evaluating and understanding the factors affecting adherence are imperative to provide insight into the population-level management of TB.

To our knowledge, there is a paucity of data on adherence to TB drugs in the general population. Although several factors such as socioeconomic factors, regimen complexity, long duration of treatment, and pattern of health care delivery system are known to affect adherence to TB drugs, the extent to which additional factor affects TB adherence needs to be explored (WHO, 2003). Past studies evaluating the adherence either focused on highly specific patient population, for instance, those with acquired immune deficiency syndrome (Shayo et al., 2015; Dhungana et al., 2019), or based on local registry data with limited sample size and lack of medical history of the individual patient (Mok et al., 2018). Therefore, we sought to assess the adherence to TB drugs and factors affecting adherence among TB patients in South Korea by using a nationwide claims database from 2017 to 2018.

## Methods

### Data Source

We used the Health Insurance Review and Assessment Service-National Patient Sample (HIRA-NPS) database from 2017 to 2018. South Korea operates a universal single-payer healthcare system, covering the entire 50 million population (Shin et al., 2011). Based on the fee-for-service system, this data contains all detailed demographic and healthcare utilization information, including all records of procedures, drugs, and diagnoses (International Classification of Diseases, 10th Revision; ICD-10 code). The database is constructed on yearly basis using age- and gender-stratified random sampling of the entire claims data, and the overall positive predictive value of diagnosis records in the claims data is 82% (Park et al., 2017).

### Study Population

**FIGURE 1:**
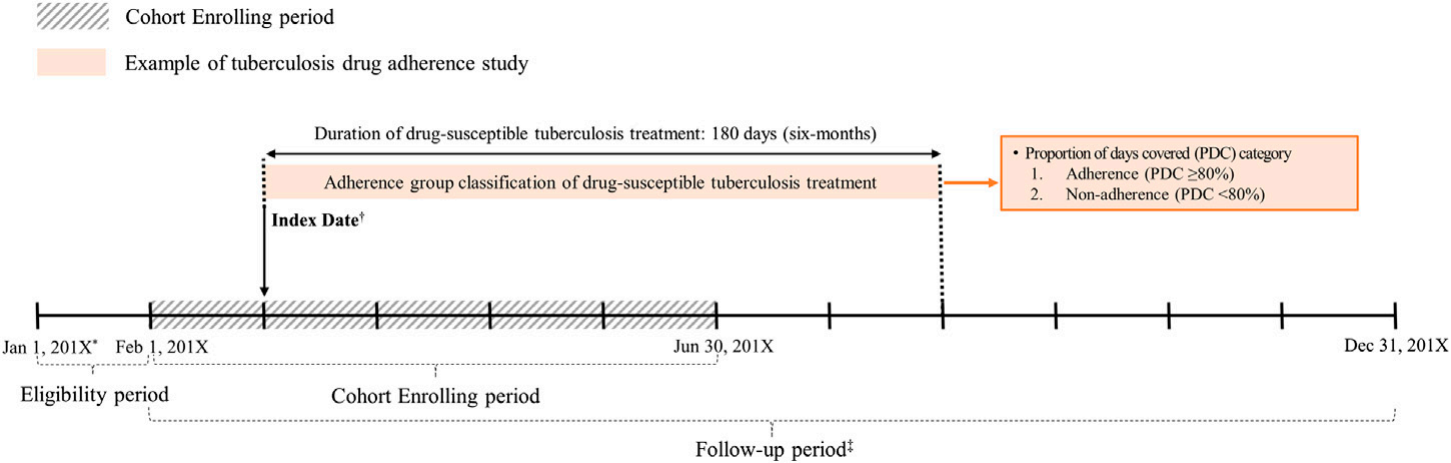
Study design of drug-susceptible tuberculosis adherence study. ^*^201X: 2017 or 2018. ^†^The first date of prescription with a quadruple or triple regimen. ^‡^Prescribed at least one of first-line tuberculosis drugs; first-line drugs for drug-susceptible tuberculosis: isoniazid, rifampicin (rifampin), rifabutin, ethambutol, pyrazinamide.

We conducted a retrospective cohort study that included drug-susceptible TB patients who received triple or quadruple regimen identified using prescription records for isoniazid, rifampicin, rifampin, ethambutol, pyrazinamide, and rifabutin, which are the drugs recommended as the first-line treatment for drug-susceptible TB (Supplementary Table S1; Pettit et al., 2018). We excluded the patients 1) with a prescription for any of the first-line drugs in January of each year to restrict our cohort to incident patients, 2) unavailable for follow-up for 180 days, or 3) did not initiate treatment with the standardized TB drug regimen recommended by the current WHO guideline (Nahid et al., 2016). The index date was defined as the first date of triple or quadruple regimen initiation, and all patients were monitored for 180 days (duration of adherence assessment) (Figure 1).

### Measure of Adherence

In this study, adherence was evaluated using the proportion of days covered (PDC), calculated as total number of days covered by a medication divided by the total number of study participation days. According to the recommended drug-susceptible TB treatment duration, each patient was followed up for 180 days from the index date (Hess et al., 2006; Nahid et al., 2016). Based on the calculated PDC, the study population was classified into two groups: 1) adherent group: patients with PDC ≥80%; 2) non-adherent group: patients with PDC <80%. The patients were considered adherent if they received at least one drug from the initially prescribed triple or quadruple regimen in the daily assessment. PDC is a conservative estimate suitable for evaluating adherence to a therapy with frequent treatment switches or involving multiple drugs as it does not include overlapping periods between prescriptions (Raebel et al., 2013). We also measured time-to-discontinuation defined as a time interval between the index date and the last date of prescription to offer additional information on adherence (Vrijens et al., 2012). Given that TB patients usually become asymptomatic following 2-months intensive phase of therapy, this may potentially lead to early discontinuation of treatment and resulting in low PDC. Therefore, we estimated time-to-discontinuation to complement PDC in assessing adherence to TB drugs.

### Potential Confounders

We assessed patient characteristics that may be related to the adherence to TB treatment. Demographic characteristics including age, sex, and type of insurance (health insurance, medical aid), type of medical institution, and regimen type (combinations type: triple and quadruple, formulation type: single and combination tablet) were assessed at the index date. We assessed comorbidities, use of comedications, and concomitant drugs as clinical characteristics by using in- and out-patient data, including prescriptions and diagnosis codes. Comorbidities (diabetes mellitus, chronic obstructive pulmonary disease, chronic liver disease, malnutrition, depression, dementia) were assessed during index year. Comedications included systemic steroids and pyridoxine, which are the drugs that may influence adherence to TB drugs. Number of concomitant drugs were defined as the maximum number of drugs in a single prescription among the overall prescriptions during the 180-days assessment periods.

### Statistical Analysis

We described the distribution of PDC in study population by decile. The median time-to-discontinuation was assessed using the Kaplan-Meier survival curve during 6 months following treatment initiation. Patients were considered to be on treatment if a gap between the end date of the previous prescription and the date of subsequent prescription is less than 15 days for the main analysis and 7 days for sensitivity analysis. Based on the adherence status, demographic characteristics, type of regimen, and clinical characteristics were summarized using counts and proportions for categorical variables. Chi-square test was used to assess differences between adherent and non-adherent groups. We used a logistic regression model to evaluate the association between adherence and relevant factors by estimating the adjusted odds ratio (aOR) and 95% confidence interval (CI). The model was adjusted for age, sex, insurance type, medical institution type, regimen type, comorbidities, comedication, and concomitant drugs. We conducted two sensitivity analysis for identifying factors associated with adherence. First, as recommended treatment duration differs between the quadruple and triple regimen, we used two different assessment periods based on the regimen type: 1) quadruple regimen: 180 days; 2) triple regimen: 270 days. Second, adherence status was ascertained using only the continuation phase of therapy (4 months) to demonstrate whether the factors were affected by the duration of assessment period. All statistical analyses were conducted using SAS 9.4 for Windows (SAS Institute Inc, Cary, NC), and a two-tailed value of *p* ≤ 0.05 was considered as statistically significant.

## Results

**FIGURE 2:**
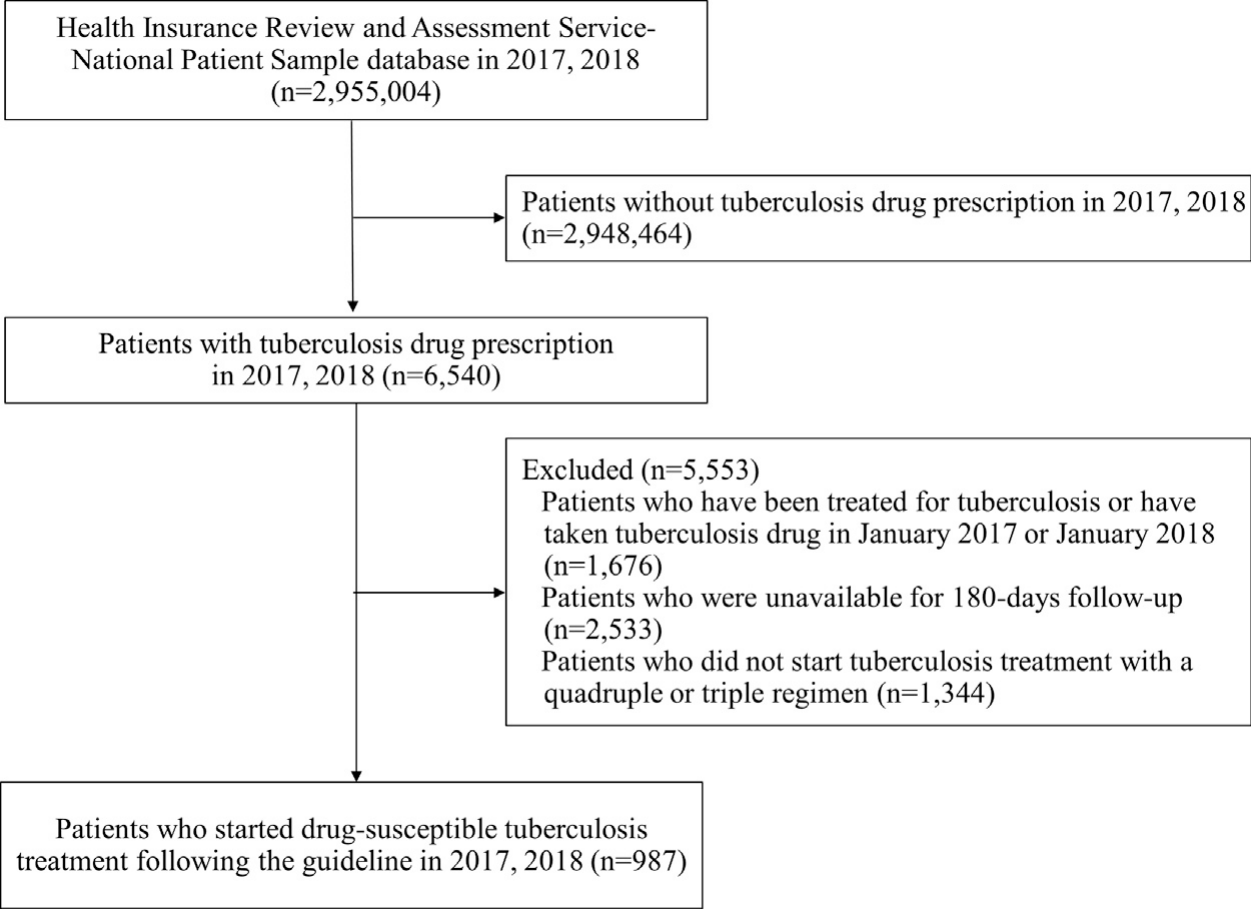
Study flowchart for patient selection of drug-susceptible tuberculosis adherence study.

**FIGURE 3:**
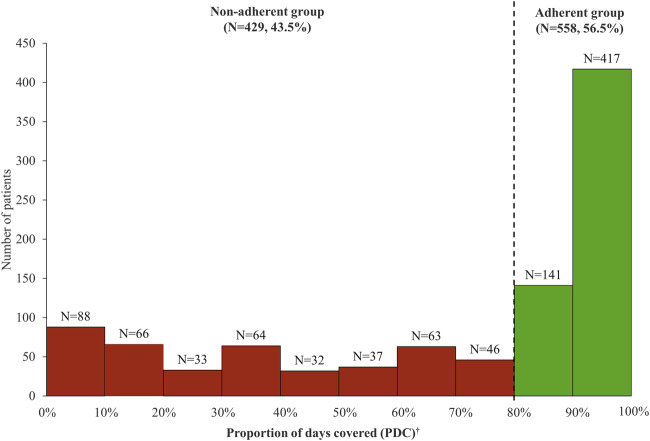
Distribution of proportion of days covered (PDC) among patients initiating tuberculosis drug. *Mean PDC was 68.87% (standard deviation, 33.37%). ^†^Patients with PDC ≥80% were classified as adherent group and patients with PDC <80% were classified as non-adherent group.

**FIGURE 4:**
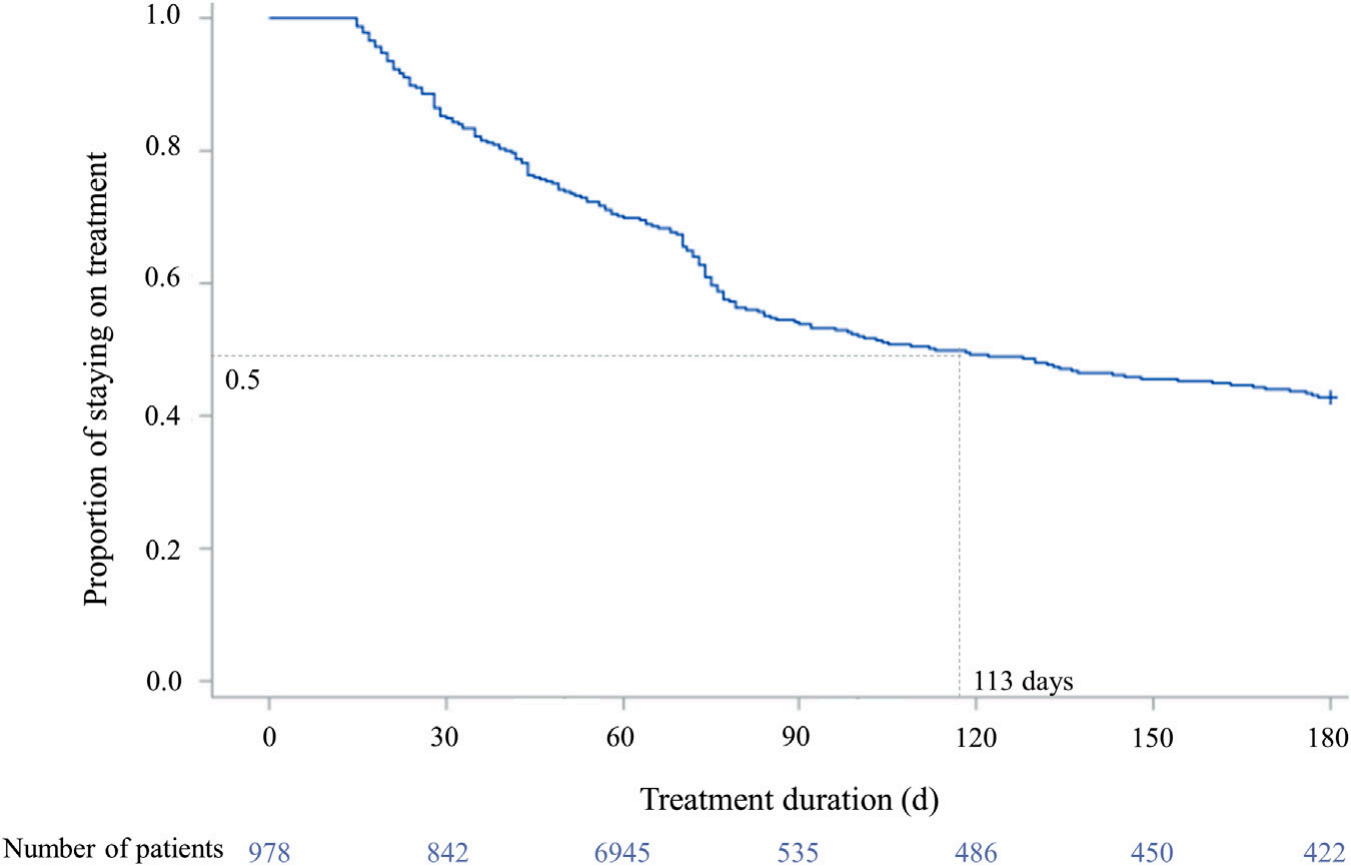
Time-to-discontinuation of tuberculosis drug in study population. ^†^Drug-susceptible tuberculosis treatment was defined as discontinued if the prescription gap was over 14 days. ^‡^The median time-to-discontinuation: 113 days (interquartile range 96–136). ^¶^Log-rank test: *p* < 0.0001.

Of 6,540 patients with prescription history for any of the first-line TB drugs, 987 patients initiated guideline-recommended standard TB treatment during the study period (Figure 2). Among them, there were 558 (56.5%) adherent and 429 (43.5%) non-adherent patients, with the overall mean PDC of 68.87% (standard deviation, SD 33.37%) (Figure 3). During the 180-days adherence assessment period, the median time-to-discontinuation was 113 days (interquartile range, IQR 96–136) (Figure 4).

In terms of baseline characteristics, adherent group had higher proportion of patients initiating quadruple regimen over triple regimen. Non-adherent group had higher proportions of the elderly, patients with medical aid and comorbidities, such as depression, dementia, diabetes mellitus, and use of systemic steroids. Moreover, the proportion of concomitant drugs (more than eight drugs use) was higher in the non-adherent group (Table 1).

**TABLE 1:** Demographic characteristic of the patients receiving tuberculosis drugs by adherence status in 2017–2018.

Covariates	Overall population (N = 987, %)	Adherence ^a^(N = 558, %)	Non-adherence ^a^(N = 429, %)	*p*-value
**Age, years**				<0.0001
Median (Q1–Q3)	62 (47–87)	57 (42–72)	67 (52–87)	
0–19	13 (1.32)	7 (1.63)	6 (1.08)	
20–34	119 (12.06)	35 (8.16)	84 (15.05)	
35–49	173 (17.53)	56 (13.05)	117 (20.97)	
50–64	260 (26.34)	100 (23.31)	160 (28.67)	
≥65	422 (42.76)	231 (53.85)	191 (34.23)	
**Sex**				0.9917
Male	598 (60.59)	338 (60.57)	260 (60.61)	
Female	389 (39.41)	220 (39.43)	169 (39.39)	
**Insurance type**				0.1402
Health insurance	890 (90.17)	510 (91.40)	380 (88.58)	
Medical aid	97 (9.83)	48 (8.60)	49 (11.42)	
**Medical institution type**				0.2605
Tertiary hospital	355 (35.97)	150 (34.97)	205 (36.74)	
General hospital	507 (51.37)	221 (51.52)	286 (51.25)	
Primary hospital	94 (9.52)	48 (11.19)	46 (8.24)	
Clinic	31 (3.14)	10 (2.33)	21 (3.76)	
**Regimen type**				<0.0001
Triple regimen (Three among the first-line drugs)^b^	186 (18.84)	56 (10.04)	130 (30.30)	
Quadruple regimen (HREZ, HEZ+Rfb, HREZ+Rfb)	801 (81.16)	502 (89.96)	299 (69.70)	
**Formulation of tablet**				0.0508
Single tablet	796 (80.65)	438 (78.49)	358 (83.45)	
Combination tablet	191 (19.35)	120 (21.51)	71 (16.55)	
**Comorbidities** ^**c**^				
Diabetes mellitus	331 (33.54)	154 (27.60)	177 (41.26)	<0.0001
Chronic obstructive pulmonary disease	320 (32.42)	180 (32.26)	140 (32.63)	0.9005
Chronic liver disease	134 (13.58)	72 (12.90)	62 (14.45)	0.4813
Malnutrition	202 (20.47)	108 (19.35)	94 (21.91)	0.3237
Depression	132 (13.37)	61 (10.93)	71 (16.55)	0.0102
Dementia	125 (12.66)	39 (6.99)	86 (20.05)	<0.0001
**Comedication** ^**d**^				
Systemic steroid	106 (10.73)	45 (8.06)	61 (14.22)	0.0020
Pyridoxine	771 (78.12)	433 (77.60)	338 (78.79)	0.6542
**Concomitant drugs** ^**d**^				0.0018
1–4	237 (24.01)	131 (23.48)	106 (24.71)	
5–8	315 (31.91)	203 (36.38)	112 (26.11)	
>8	435 (44.07)	224 (40.14)	211 (49.18)	

H, isoniazid; R, rifampicin(rifampin); E, ethambutol; Z, pyrazinamide; Rfb, rifabutin; cOR, crude odds ratio; aOR, adjusted odds ratio; CI, confidence interval.

^a^Adherence to tuberculosis drugs defined as proportion of days covered (PDC) ≥80%, non-adherence to tuberculosis drugs defined as proportion of days covered <80%.

^b^ First-line drugs for drug-susceptible tuberculosis: isoniazid, rifampicin (rifampin), ethambutol, pyrazinamide, rifabutin.

^c^ Comorbidities were assessed during index year.

^d^Comedications and concomitant drugs were assessed during 180-days adherence assessment period.

Results from the logistic regression analysis show that, compared with triple regimen, quadruple regimen was more likely to be associated with adherence (aOR 4.14; 95% CI 2.78–6.17). Non-adherence was associated with the elderly patients (aged 65 years or older) (aOR 0.53; 95% CI 0.35–0.81) and those with dementia (aOR 0.53; 95% CI 0.34–0.85) or with diabetes mellitus (aOR 0.70; 95% CI 0.52–0.96). Meanwhile, a number of concomitant drugs or fixed-dose combinations (FDCs) pills were not associated with adherence (Table 2).

**TABLE 2:** Logistic regression analysis on the association between population characteristics and adherence to the tuberculosis drugs in 2017–2018.

Covariates	cOR (95% CI)	aOR ^a^(95% CI)
**Age, years**		
0–19	0.41 (0.13–1.28)	0.50 (0.15–1.71)
20–34	1.15 (0.69–1.91)	1.04 (0.61–1.77)
35–49	1.00 (Reference)	1.00 (Reference)
50–64	0.77 (0.51–1.15)	0.83 (0.54–1.29)
≥65	0.40 (0.27–0.57)	0.53 (0.35–0.81)
**Sex**		
Male	1.00 (Reference)	1.00 (Reference)
Female	1.00 (0.77–1.30)	1.06 (0.79–1.41)
**Insurance type**		
Health insurance	1.00 (Reference)	1.00 (Reference)
Medical aid	0.73 (0.48–1.11)	0.78 (0.50–1.24)
**Medical institution type**		
Tertiary hospital	1.00 (Reference)	1.00 (Reference)
General hospital	0.95 (0.72–1.25)	0.97 (0.72–1.30)
Primary hospital	0.70 (0.44–1.11)	0.74 (0.45–1.22)
Clinic	1.54 (0.70–3.36)	1.23 (0.52–2.88)
**Regimen type**		
Triple regimen (Three among the first-line drugs)^b^	1.00 (Reference)	1.00 (Reference)
Quadruple regimen (HREZ, HEZ+Rfb, HREZ+Rfb)	3.90 (2.76–5.50)	4.14 (2.78–6.17)
**Formulation of tablet**		
Single tablet	1.00 (Reference)	1.00 (Reference)
Combination tablet	1.38 (1.00–1.91)	1.04 (0.71–1.53)
**Comorbidities** ^**c**^		
Diabetes mellitus	0.54 (0.42–0.71)	0.70 (0.52–0.96)
Chronic obstructive pulmonary disease	0.98 (0.75–1.29)	1.21 (0.89–1.64)
Chronic liver disease	0.88 (0.61–1.26)	1.06 (0.71–1.58)
Malnutrition	0.86 (0.63–1.17)	0.95 (0.67–1.34)
Depression	0.62 (0.43–0.89)	0.89 (0.58–1.35)
Dementia	0.30 (0.20–0.45)	0.53 (0.34–0.85)
**Comedication** **^d^**		
Systemic steroid	0.53 (0.35–0.80)	0.64 (0.41–1.02)
Pyridoxine	0.93 (0.69–1.27)	0.98 (0.68–1.41)
**Concomitant drugs** **^d^**		
1–4	1.00 (Reference)	1.00 (Reference)
5–8	1.47 (1.04–2.07)	1.27 (0.81–1.98)
>8	0.86 (0.63–1.18)	0.92 (0.59–1.41)

H, isoniazid; R, rifampicin (rifampin); E, ethambutol; Z, pyrazinamide; Rfb, rifabutin; cOR, crude odds ratio; aOR, adjusted odds ratio; CI, confidence interval.

^a^ Multivariable logistic regression was fully adjusted with all potential confounders.

^b^ First-line drugs for drug-susceptible tuberculosis: isoniazid, rifampicin (rifampin), ethambutol, pyrazinamide, rifabutin.

^c^ Comorbidities were assessed during index year.

^d^ Comedications and concomitant drugs were assessed during 180-days adherence assessment period.

We conducted sensitivity analysis using different treatment durations based on the regimen type (i.e., 6 months for quadruple regimen, and 9 months for triple regimen) to measure adherence and identify the associated factors. There were 258 (42.3%) adherent and 352 (57.7%) non-adherent patients, with the overall mean PDC of 62% (SD 33%) (Supplementary Figure S1). Consistent with our main result, the quadruple regimen was more likely to be associated with adherence compared with the triple regimen (aOR 6.48; 95% CI 3.71–11.33) (Supplementary Table S2). This trend was also observed when we restricted our adherence assessment period to the continuation phase of therapy, with aOR of 6.48 (95% CI 3.71–11.33) for quadruple regimen.

## Discussion

In this study, we included 987 patients newly initiated on the drug-susceptible TB treatment, in which 56.5% of the patients were adherent (PDC ≥80%) during the entire study period. The median time-to-discontinuation was 113 days in the study population. The factors such as older age, history of dementia, and diabetes mellitus were associated with non-adherence. Compared with the patients who initiated the triple regimen, the patients with the quadruple regimen were more likely to adhere to the drug. However, pyridoxine use or the number of concomitant drugs were not associated with adherence to TB drugs.

Our findings are consistent with other TB drug adherence studies. In a randomized clinical trial using the directly observed therapy (DOT) method, the adherence was 75.1% (DOT: 751 per 1,000 cases) (Karumbi and Garner, 2015). One study in Brazil involved 745 TB patients and demonstrated a low proportion of TB treatment adherence in patients aged ≥60 years (58.5%). This result was largely consistent with our study (proportion of adherence to TB treatment 53.9% in patients aged ≥65 years). Low treatment adherence in the elderly may be due to the higher risk of multi-morbidity and subsequent polypharmacy risk among this population, which may lead to a non-adherence to drugs compared to the younger population (Roy et al., 2017; Freire et al., 2019). In the registry-based studies in South Korea and Spain, the adherences were also similar to our main result (South Korea, 63.7%; Spain, 69.1%) (Ambrona de Marcos et al., 2018; Mok et al., 2018). However, given the inherent limitations of DOT trials or registry data, the association between medical use and adherence could not be assessed in previous studies.

The previous studies suggested that the elderly patients with chronic medical conditions were more likely to adhere to the prescribed therapy (WHO, 2003; Mantarro et al., 2015; Rashid et al., 2015; Chang et al., 2019). However, in our study, adherence to TB drugs was less common among those aged 65 or older. This finding may be attributed to the prevalence of dementia and concomitant drug use among elderly patients included in our study. Specifically, poor adherence to a pharmacological therapy was more common in the dementia population (WHO, 2003), where forgetfulness was a major barrier to adherence according to qualitative research (Cramer, 1991). Moreover, dementia was more prevalent in our TB patients aged ≥65 years than in general elderly Korean population (27.5 vs. 9.2%), and there was a notable difference in the proportion of dementia between adherent and non-adherent groups (18.9 vs. 34.6%) in patients aged ≥65 years (Ministry of Health and Welfare, 2013). Therefore, we believe relatively high prevalence of dementia in our study population may have driven the non-adherence to TB drugs. Also, the use of multiple drugs is a well-recognized problem for elderly patients, which may likely lead to non-adherence to drugs due to the dosing schedule complexity and high pill burden (WHO, 2003; Marcum and Gellad, 2012).

According to the TB treatment guidelines, offending drugs are required to be discontinued or switched to second-line drugs in case of adverse drug reactions (ADRs) such as gastrointestinal upset, rash, hepatoxicity, or optic neuritis (Nahid et al., 2016; WHO, 2017). Indeed, complications during active treatment would negatively impact adherence, and our study also showed that triple regimen, history of diabetes mellitus, and systemic steroid use during treatment were the factors associated with non-adherence. Triple regimen is generally recommended for patients with vulnerable health conditions such as hepatic or renal disease who are at higher risk for ADRs from TB drugs. Therefore, those initiating triple regimen may represent a subset of the study population who are more prone to temporarily hold treatment in case of the ADRs. Diabetes mellitus is a risk factor for poor prognosis from TB, and thus patients with diabetes mellitus may be more susceptible to TB complications leading to intervention in active treatment (WHO, 2018). Furthermore, given that systemic steroids are indicated for the management of various complications or ADRs from TB drugs, such use is associated with poor adherence to TB drugs (Nahid et al., 2016). Of note in the context of ADRs from TB drugs, we observed a high proportion of pyridoxine use regardless of adherence status. Pyridoxine is usually co-prescribed with isoniazid to prevent peripheral neuropathy. While concomitant use of pyridoxine and adherence showed null association, it is reassuring to note that the majority of patients were receiving guideline-recommended treatment.

As the number of pills required for drug-susceptible TB treatment is one of the major barriers to adherence, fixed-dose combinations (FDCs) are commonly used and recommended by WHO. Owing to anticipated advantages of FDCs, we initially hypothesized that the use of FDCs would improve patient adherence. However, our study did not show significant association between the use of FDCs and adherence, and only 20% of our study population utilized FDCs for TB treatment. In fact, compared with a single-drug formulation, FDCs did not show any additional benefit in TB treatment outcomes, which implies a lack of evidence to change the prescribing patterns of physicians (Schiff et al., 2011; Albanna et al., 2013; Gallardo et al., 2016). Nevertheless, it is without a doubt that FDCs reduce pill burden on the patients’ perspective, which may correlate with adherence. While our study did not identify any association between the FDCs and adherence, further studies are needed to build upon our finding to ascertain the role of FDCs on the adherence and TB treatment outcome.

To our knowledge, this is the first observational study that assessed adherence to TB drugs using administrative claims data to capture all inpatient and outpatient medication records. Thus, we were able to control recall bias arising from any distortion by patients or health care professionals. Also, as our sample database represents the entire 50 million South Korean population, our findings serve as the real-world data for the public health stakeholders in implementing an effective TB eradication strategy. Finally, we used PDC, instead of medication possession ratio (MPR), to determine an individual’s adherence to the treatment. While both are commonly used, PDC is a more conservative approach in measuring adherence as the overlaps between prescriptions are not included in the calculation. However, it should also be noted that PDC may underestimate adherence due to the inclusion of the time period after discontinuation in the calculation (Hess et al., 2006).

Our study also has several limitations. First, we could not ascertain if the patients actually had received or consumed the prescribed drugs, which in turn could lead to overestimation of adherence. Apart from lack of ascertainment on actual consumption of TB drugs for each patient, we used a conservative adherence measure, PDC, to minimize overestimating adherence. Kaplan-Meier plot with median time-to-discontinuation was presented to complement PDC in assessing adherence, in which we identified early discontinuation of TB drugs. Second, we used the 180-days adherence assessment period to ensure enough sample size, which inadvertently resulted in not fully covering the recommended treatment duration for the triple regimen. However, we conducted a sensitivity analysis using the recommended treatment duration (i.e., 6 months for quadruple regimen; 9 months for triple regimen) as an adherence assessment period and demonstrated similar findings with the main analysis. Additionally, we conducted a sensitivity analysis on the treatment adherence during continuation phase (2 months after the TB treatment initiation) to determine the impact of asymptomatic TB on adherence status. The results obtained for the continuation phase were also consistent with our main findings. Thus, we believe that the adherence assessment period did not change the direction of the relationship between the regimen and adherence. Third, our adherence definition may have overestimated adherence. However, multi-drug combination therapy (e.g., HRZE) was used more than isoniazid, rifampicin alone during four months of the continuation phase. Single drug use was only 91 cases (2.9%) among 3,144 overall prescriptions related to TB medications in this study. Moreover, our adherence definition is equal to an evaluation indicator for national tuberculosis treatment guidelines of South Korea (Health Insurance Review and Assessment Service, 2019). Fourth, death, the severity of the disease, adverse events, and dynamics of the underlying medical conditions may have led to non-adherence to the prescribed drugs, but each individual’s detailed clinical assessment could not be captured in the claims data. Fifth, residual confounding could remain as factors related to socioeconomics or healthcare system affecting adherence, including marital status, income level, the behavior of healthcare service providers cannot be determined from the claims database (Mathes et al., 2014). Lastly, since we included both in- and out-patient data, the number of concomitant drugs may be overestimated, considering inpatient treatment’s close monitoring setting.

In conclusion, our study observed that approximately half of incident TB patients were non-adherent and provided epidemiological insight into the factors related to adherence to the TB drugs. These are important concerns for physicians when managing infectious diseases, as well as for authorities when establishing the public health policies on ending the TB epidemic. In order to achieve WHO-recommended TB treatment success rate of ≥90%, interdisciplinary efforts from multiple stakeholders are imperative to address the low adherence rate observed in our study. Further studies are warranted to implement an effective strategy to reinforce drug adherence during active TB infection.

## Data Availability

The data analyzed in this study is subject to the following licenses/restrictions: Our study used Health Insurance Review and Assessment Service-National Patient Sample (HIRA-NPS) database. HIRA forbids the transfer, rent, or sale of the database to any third party other than the researcher, who obtained the approval for the provided database. Requests to access these datasets should be directed to HIRA; Official website of HIRA: https://opendata.hira.or.kr; Contact information of data access committee: +82-33-739-1083.
