# Supplementary material for: Adherence and Associated Factors of Treatment Regimen in Drug-Susceptible Tuberculosis Patients
Source: Front Pharmacol. 2021 Mar 15;12:625078. doi: 10.3389/fphar.2021.625078 (PMC8005597; doi:10.3389/fphar.2021.625078)
Supplement: Supplementary file 1 [file datasheet1.docx]

Supplementary Material

**Figure S1.** Distribution of proportion of days covered (PDC) in patients initiating tuberculosis drugs (sensitivity analysis for 9months follow-up of triple regimen initiated patients)


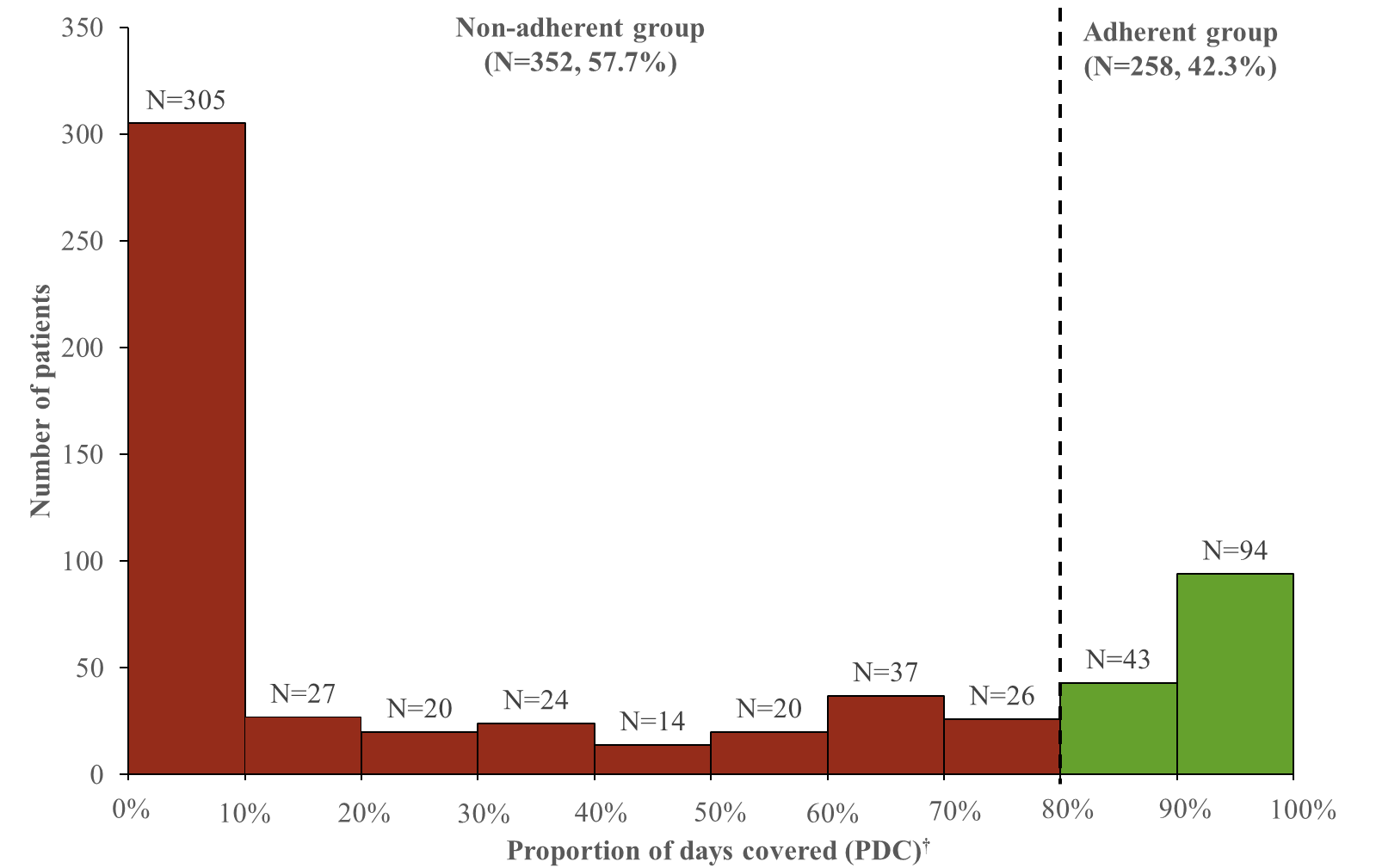


^*^Mean PDC was 62% ± 33%

^†^Patients with Proportion of days covered (PDC) ≥80% were classified as adherence and patients with PDC <80% were classified as non-adherence

**Figure S2. Time-to-discontinuation of tuberculosis drug in overall population (sensitivity analysis: grace period 7 days)**

**
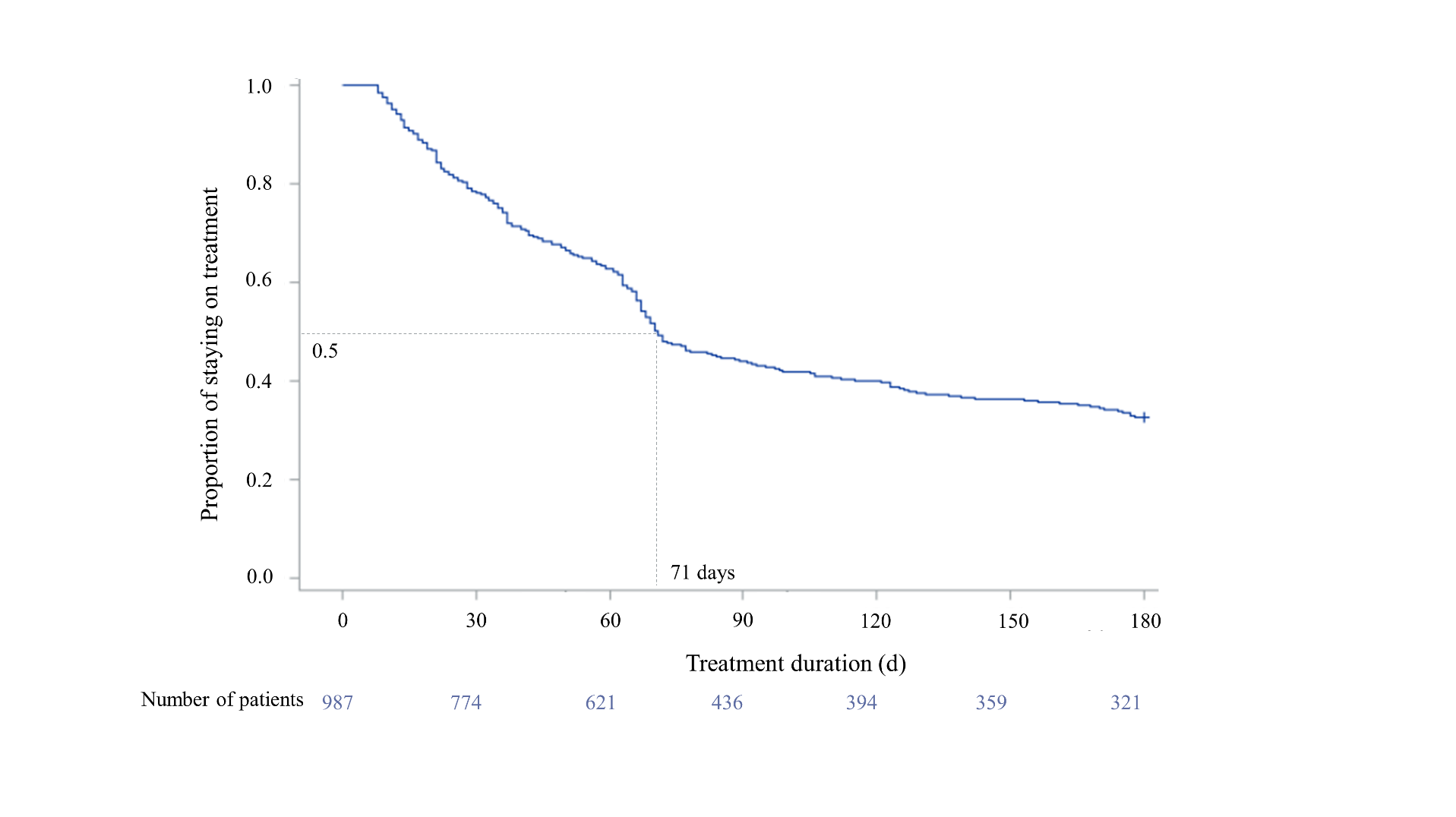
**

^*^Patients with proportion of days covered (PDC) ≥80% were classified as adherence and patients with PDC <80% were classified as non-adherence

^†^Drug-susceptible tuberculosis treatment was defined as discontinued if the prescription gap was over 7 days

^‡^ The median time-to-discontinuation: 71 days (IQR 68-77)

**Table S1.** Drug list for drug-susceptible tuberculosis adherence study

| **Treatment class** | **Ingredient name** | **ATC code** |
| --- | --- | --- |
| First-line group | Isoniazid (INH) | J04AC01 |
|  | Rifampicin (RIF) | J04AB02 |
|  | Ethambutol (EMB) | J04AK02 |
|  | Pyrazinamide (PZA) | J04AK01 |
|  | Rifabutin (RFB) | J04AB04 |
|  | Combination (INH, PZA, RIF) | J04AM05 |
|  | Combination (INH, RIF, PZA, EMB) | J04AM06 |
|  | Combination (INH, RIF) | J04AM02 |

| **Covariates** | | **cOR (95% CI)** | **aOR (95% CI)^¶^** |
| --- | --- | --- | --- |
| **Age, years** | |  |  |
|  | 0-19 | 0.55 (0.13-2.32) | 0.66 (0.14-3.15) |
|  | 20-34 | 1.11 (0.61-2.02) | 1.02 (0.53-1.97) |
|  | 35-49 | 1.00 (Reference) | 1.00 (Reference) |
|  | 50-64 | 1.09 (0.68-1.77) | 1.12 (0.66-1.92) |
|  | ≥ 65 | 0.57 (0.37-0.89) | 0.64 (0.38-1.09) |
| **Sex** | |  |  |
|  | Male | 1.00 (Reference) | 1.00 (Reference) |
|  | Female | 1.06 (0.76-1.47) | 1.15 (0.80-1.67) |
| **Insurance type** | |  |  |
|  | Health insurance | 1.00 (Reference) | 1.00 (Reference) |
|  | Medical aid | 0.78 (0.44-1.37) | 0.8 (0.43-1.49) |
| **Medical institution type** | |  |  |
|  | Tertiary hospital | 1.00 (Reference) | 1.00 (Reference) |
|  | General hospital | 1.09 (0.77-1.55) | 1.10 (0.75-1.62) |
|  | Primary hospital | 0.94 (0.53-1.67) | 0.89 (0.46-1.69) |
|  | Clinic | 0.82 (0.31-2.16) | 0.53 (0.18-1.52) |
| **Regimen type** | |  |  |
|  | Triple regimen (Three among the first-line drugs)^†^ | 1.00 (Reference) | 1.00 (Reference) |
|  | Quadruple regimen (HREZ, HEZ+Rfb, HREZ+Rfb) | 6.45 (3.92-10.59) | 6.48 (3.71-11.33) |
| **Formulation of tablet** | |  |  |
|  | Single tablet | 1.00 (Reference) | 1.00 (Reference) |
|  | Combination tablet | 1.47 (0.97-2.21) | 1.00 (0.61-1.62) |
| **Comorbidities**^¶^ | |  |  |
|  | Diabetes | 0.65 (0.46-0.92) | 0.75 (0.50-1.14) |
|  | Chronic obstructive pulmonary disease | 1.22 (0.86-1.72) | 1.42 (0.96-2.12) |
|  | Chronic liver disease | 0.8 (0.5-1.28) | 0.92 (0.55-1.56) |
|  | Malnutrition | 1.12 (0.75-1.68) | 1.06 (0.68-1.67) |
|  | Depression | 0.56 (0.34-0.91) | 0.62 (0.36-1.09) |
|  | Dementia | 0.43 (0.25-0.73) | 0.74 (0.39-1.39) |
| **Comedication^§^** | |  |  |
|  | Systemic steroid | 0.6 (0.34-1.07) | 0.52 (0.28-0.98) |
|  | Pyridoxine | 1.23 (0.84-1.82) | 1.04 (0.65-1.67) |
| **Concomitant drugs^§^** | |  |  |
|  | 1-4 | 1.00 (Reference) | 1.00 (Reference) |
|  | 5-8 | 2.04 (1.32-3.14) | 1.37 (0.78-2.41) |
|  | >8 | 1.54 (1.01-2.34) | 1.12 (0.65-1.96) |

**Table S2.** Sensitivity analysis on association between population characteristics and adherence to the tuberculosis treatment according to regimen difference (quadruple regimen: 180 days; triple regimen: 270 days)

Abbrevations: H, isoniazid; R, rifampicin (rifampin); E, ethambutol; Z, pyrazinamide; Rfb, rifabutin; cOR, crude odds ratio; aOR, adjusted odds ratio; CI, confidence interval.

^*^Adherence to tuberculosis drugs defined as proportion of days covered (PDC) ≥80%, non-adherence to tuberculosis drugs defined as proportion of days covered <80%

^†^First-line drugs for drug-susceptible tuberculosis: isoniazid, rifampicin (rifampin), ethambutol, pyrazinamide, rifabutin

^§^Comedications and concomitant drugs were assessed during 180-day adherence assessment period

^¶^Comorbidities were assessed during index year

^‡^Multivariable logistic regression was fully adjusted with all potential confounders

**Table S3.** Sensitivity analysis on the association between population characteristics and adherence to the tuberculosis treatment during continuous phase

| **Covariates** | | **cOR (95% CI)** | **aOR (95% CI)^¶^** |
| --- | --- | --- | --- |
| **Age, years** | |  |  |
|  | 0-19 | 0.38 (0.09-1.59) | 0.46 (0.10-2.22) |
|  | 20-34 | 0.82 (0.42-1.63) | 0.75 (0.36-1.58) |
|  | 35-49 | 1.00 (Reference) | 1.00 (Reference) |
|  | 50-64 | 1.07 (0.59-1.96) | 1.02 (0.53-1.96) |
|  | ≥ 65 | 0.62 (0.36-1.06) | 0.59 (0.32-1.11) |
| **Sex** | |  |  |
|  | Male | 1.00 (Reference) | 1.00 (Reference) |
|  | Female | 0.91 (0.63-1.33) | 1.10 (0.72-1.68) |
| **Insurance type** | |  |  |
|  | Health insurance | 1.00 (Reference) | 1.00 (Reference) |
|  | Medical aid | 1.27 (0.63-2.55) | 1.65 (0.76-3.61) |
| **Medical institution type** | |  |  |
|  | Tertiary hospital | 1.00 (Reference) | 1.00 (Reference) |
|  | General hospital | 0.98 (0.65-1.45) | 0.93 (0.602-1.435) |
|  | Primary hospital | 0.95 (0.48-1.86) | 0.93 (0.44-1.97) |
|  | Clinic | 2.67 (0.61-11.63) | 2.53 (0.53-12.04) |
| **Regimen type** | |  |  |
|  | Triple regimen (Three among the first-line drugs)^†^ | 1.00 (Reference) | 1.00 (Reference) |
|  | Quadruple regimen (HREZ, HEZ+Rfb, HREZ+Rfb) | 6.77 (4.44-10.33) | 6.40 (3.86-10.61) |
| **Formulation of tablet** | |  |  |
|  | Single tablet | 1.00 (Reference) | 1.00 (Reference) |
|  | Combination tablet | 1.83 (1.08-3.10) | 1.08 (0.59-2.00) |
| **Comorbidities**^¶^ | |  |  |
|  | Diabetes | 0.92 (0.62-1.37) | 1.03 (0.64-1.65) |
|  | Chronic obstructive pulmonary disease | 1.63 (1.06-2.50) | 1.71 (1.06-2.76) |
|  | Chronic liver disease | 0.85 (0.50-1.44) | 0.94 (0.52-1.69) |
|  | Malnutrition | 1.10 (0.68-1.76) | 1.02 (0.61-1.72) |
|  | Depression | 0.75 (0.44-1.27) | 0.78 (0.42-1.45) |
|  | Dementia | 0.44 (0.26-0.75) | 0.59 (0.31-1.16) |
| **Comedication^§^** | |  |  |
|  | Systemic steroid | 0.92 (0.37-2.26) | 0.63 (0.23-1.74) |
|  | Pyridoxine | 1.21 (0.81-1.80) | 1.15 (0.73-1.80) |
| **Concomitant drugs^§^** | |  |  |
|  | 1-4 | 1.00 (Reference) | 1.00 (Reference) |
|  | 5-8 | 1.51 (0.95-2.40) | 0.93 (0.52-1.66) |
|  | >8 | 1.78 (1.13-2.81) | 1.20 (0.66-2.16) |

Abbrevations: H, isoniazid; R, rifampicin (rifampin); E, ethambutol; Z, pyrazinamide; Rfb, rifabutin; cOR, crude odds ratio; aOR, adjusted odds ratio; CI, confidence interval.

^*^Adherence to tuberculosis drugs defined as proportion of days covered (PDC) ≥80%, non-adherence to tuberculosis drugs defined as proportion of days covered <80%

^†^First-line drugs for drug-susceptible tuberculosis: isoniazid, rifampicin (rifampin), ethambutol, pyrazinamide, rifabutin

^§^Comedications and concomitant drugs were assessed during 180-day adherence assessment period

^¶^Comorbidities were assessed during index year

^‡^Multivariable logistic regression was fully adjusted with all potential confounders
