# Supplementary figures and images for: Adherence and Associated Factors of Treatment Regimen in Drug-Susceptible Tuberculosis Patients
Source: Front Pharmacol. 2021 Mar 15;12:625078. doi: 10.3389/fphar.2021.625078 (PMC8005597; doi:10.3389/fphar.2021.625078)

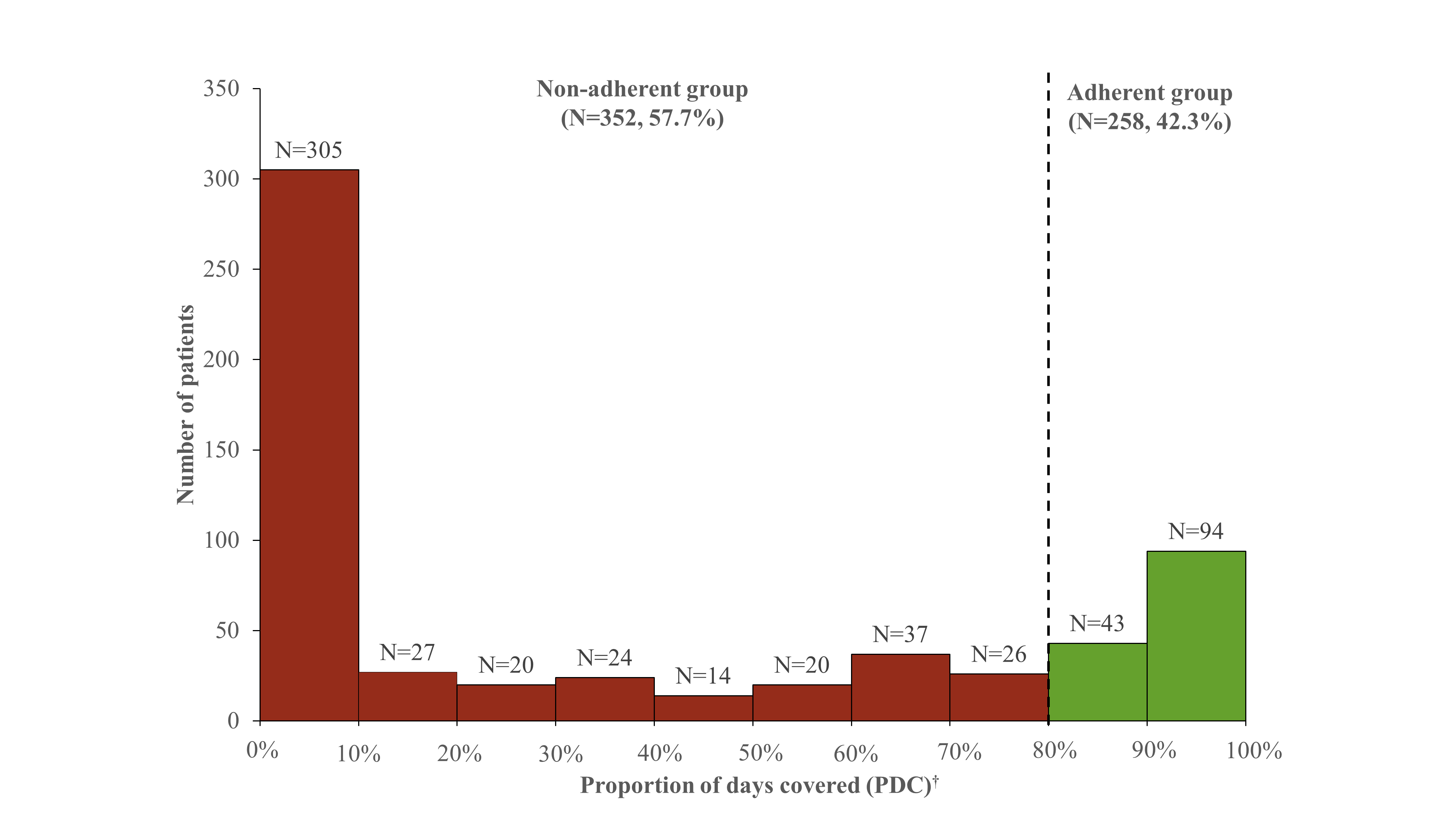

Supplement: Supplementary file 2 [file image1.tif]

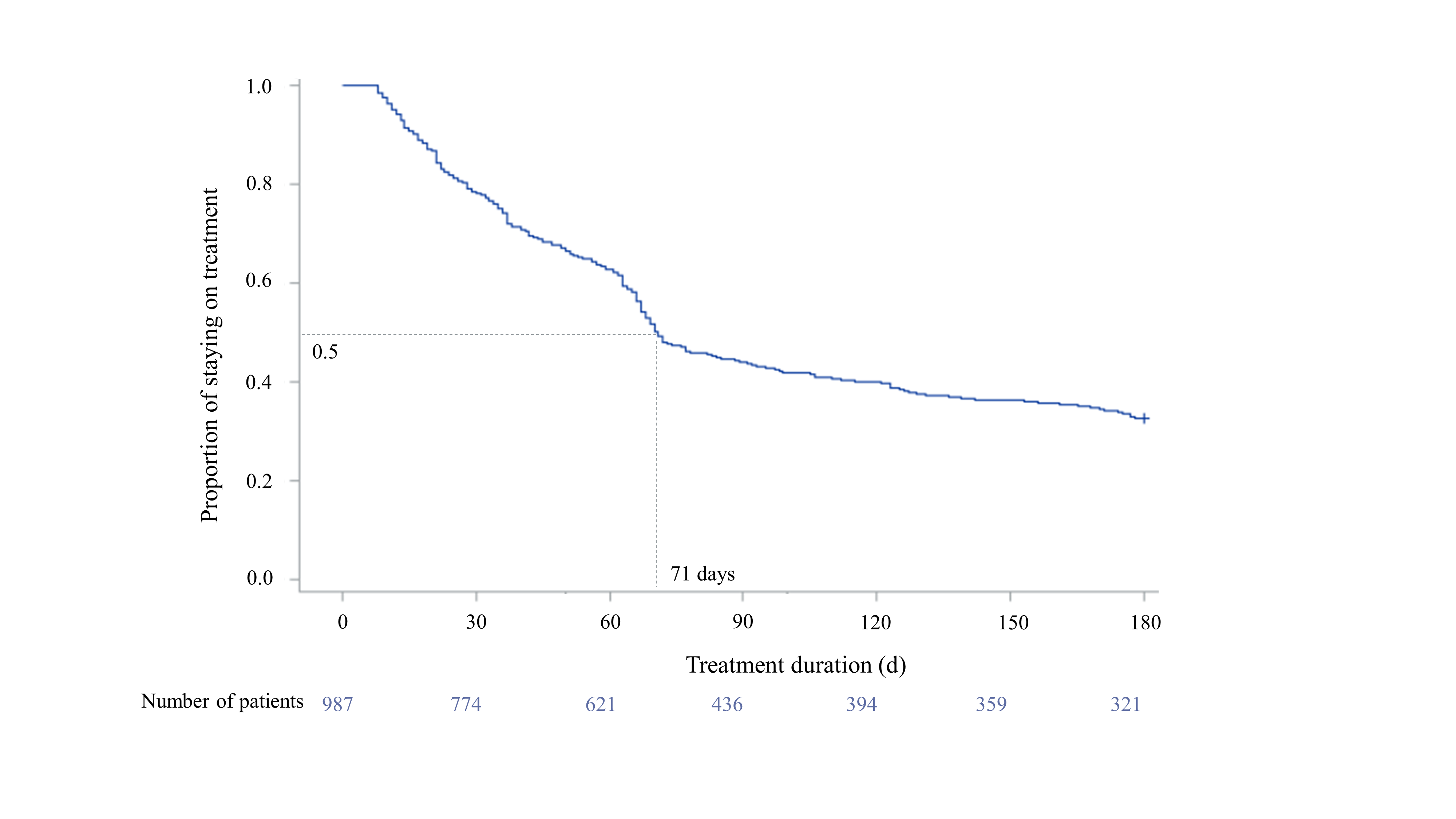

Supplement: Supplementary file 3 [file image2.tif]
